# Mapping of a N-terminal α-helix domain required for human PINK1 stabilization, Serine228 autophosphorylation and activation in cells

**DOI:** 10.1098/rsob.210264

**Published:** 2022-01-19

**Authors:** Poonam Kakade, Hina Ojha, Olawale G. Raimi, Andrew Shaw, Andrew D. Waddell, James R. Ault, Sophie Burel, Kathrin Brockmann, Atul Kumar, Mohd Syed Ahangar, Ewelina M. Krysztofinska, Thomas Macartney, Richard Bayliss, Julia C. Fitzgerald, Miratul M. K. Muqit

**Affiliations:** ^1^ MRC Protein Phosphorylation and Ubiquitylation Unit, University of Dundee, Dundee DD1 5EH, UK; ^2^ Division of Gene Regulation and Expression, School of Life Sciences, University of Dundee, Dundee DD1 5EH, UK; ^3^ Astbury Centre for Structural Molecular Biology, School of Molecular and Cellular Biology, Faculty of Biological Sciences, University of Leeds, Leeds LS2 9JT, UK; ^4^ Hertie Institute for Clinical Brain Research, University of Tübingen, Tübingen, Germany; ^5^ The German Centre for Neurodegenerative Diseases (DZNE), Tübingen, Germany; ^6^ Astex Pharmaceuticals, 436 Cambridge Science Park, Milton Road, Cambridge CB4 0QA, UK

**Keywords:** PINK1, kinase, mitochondria, translocase, phosphorylation, Parkinson's disease

## Abstract

Autosomal recessive mutations in the *PINK1* gene are causal for Parkinson's disease (PD). PINK1 encodes a mitochondrial localized protein kinase that is a master-regulator of mitochondrial quality control pathways. Structural studies to date have elaborated the mechanism of how mutations located within the kinase domain disrupt PINK1 function; however, the molecular mechanism of PINK1 mutations located upstream and downstream of the kinase domain is unknown. We have employed mutagenesis studies to define the minimal region of human PINK1 required for optimal ubiquitin phosphorylation, beginning at residue Ile111. Inspection of the AlphaFold human PINK1 structure model predicts a conserved N-terminal α-helical extension (NTE) domain forming an intramolecular interaction with the C-terminal extension (CTE), which we corroborate using hydrogen/deuterium exchange mass spectrometry of recombinant insect PINK1 protein. Cell-based analysis of human PINK1 reveals that PD-associated mutations (e.g. Q126P), located within the NTE : CTE interface, markedly inhibit stabilization of PINK1; autophosphorylation at Serine228 (Ser228) and Ubiquitin Serine65 (Ser65) phosphorylation. Furthermore, we provide evidence that NTE and CTE domain mutants disrupt PINK1 stabilization at the mitochondrial Translocase of outer membrane complex. The clinical relevance of our findings is supported by the demonstration of defective stabilization and activation of endogenous PINK1 in human fibroblasts of a patient with early-onset PD due to homozygous PINK1 Q126P mutations. Overall, we define a functional role of the NTE : CTE interface towards PINK1 stabilization and activation and show that loss of NTE : CTE interactions is a major mechanism of PINK1-associated mutations linked to PD.

## Graphical abstract

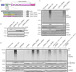

## 1. Introduction

Autosomal recessive mutations in PTEN-induced kinase 1 (PINK1) are associated with early-onset Parkinson's disease (PD) [1]. Human PINK1 (hPINK1) encodes a 581 amino acid Ser/Thr protein kinase containing a N-terminal mitochondrial-targeting sequence (MTS) (residues 1–34): catalytic kinase domain containing three major loop insertions (residues 156–510) and a C-terminal extension (CTE) (residues 511–581) [2]. Under basal cellular conditions, PINK1 is imported into mitochondria via its MTS whereupon it undergoes consecutive cleavage by mitochondrial-processing protease and the rhomboid protease PARL. The N-terminally cleaved PINK1 fragment (residues Phe104-end) subsequently undergoes 20S proteasomal degradation via the N-end rule pathway [3].

Upon mitochondrial depolarization, which can be induced by mitochondrial uncouplers (e.g. Antimycin A/Oligomycin (A/O) or carbonyl cyanide m-chlorophenyl hydrazone (CCCP)), full-length human PINK1 protein is stabilized at the mitochondria where it becomes autophosphorylated and active [4]. Active PINK1 phosphorylates ubiquitin at Serine65 (Ser65) that recruits the Parkinson's linked ubiquitin ligase, Parkin, to the mitochondrial surface whereupon it phosphorylates Parkin at an equivalent Ser65 residue within its N-terminal ubiquitin-like (Ubl) domain leading to activation of Parkin E3 ligase activity and ubiquitin-dependent elimination of damaged mitochondria by autophagy (mitophagy) [5–7]. Active PINK1 also indirectly induces the phosphorylation of a subset of Rab GTPases including Rab 8A at a highly conserved Serine residue (Ser111) that lies within the RabSF3 motif [8].

Structural and biochemical analysis of catalytic domain-containing fragments of insect orthologues of PINK1, namely, *Tribolium castaneum* (Tc) [PDB 5OAT] and *Pediculus humanus corporis* (Phc) (in complex with ubiquitin via a nanobody) [PDB 6EQI], have shed light on intrinsic kinase domain-mediated mechanisms of PINK1 activation [9,10]. These studies identified a critical role of autophosphorylation of a Serine residue (TcPINK1, Ser205; PhcPINK1 Ser202) within the N-lobe for activation and ubiquitin substrate recognition that is equivalent to Ser228 of hPINK1 [9,10]. Structural analysis also revealed a previously unidentified role for the third loop insertion (INS3) towards substrate recognition and catalytic activity [9,10]. Mutagenesis studies of human PINK1 in cells has confirmed conservation of these mechanisms; however, unambiguous detection of PINK1 autophosphorylation at Ser228 (equivalent to the insect pSer205/202) is outstanding.

The structures have provided atomic insights into the pathogenic mechanism of key human PINK1 disease-associated mutations that lie within the kinase domain. However, a number of disease mutations lie outside the kinase domain within the upstream N-terminal region of PINK1 and current published structures provide no insights into its function [9,10]. Upon mitochondrial depolarization, full-length human PINK1 accumulates on the outer mitochondrial membrane (OMM) in association with the Translocase of outer membrane (TOM) complex; however, the molecular basis of how human PINK1 is stabilized and activated at the TOM complex remains to be fully elucidated [11,12].

Herein, we have mapped the minimal region of human PINK1, required for optimal ubiquitin phosphorylation, beginning at residue Ile111. Bioinformatic analysis of the region spanning Ile111 and the kinase domain and inspection of a predicted structural model of human PINK1 by AlphaFold [13] reveals a N-terminal α-helical extension (NTE) domain which forms intramolecular contacts with the CTE. Mutagenesis of key residues at the NTE–CTE interface indicates a critical role of this interface towards human PINK1 stabilization at the TOM complex: Ser228 autophosphorylation and PINK1 activation in cells.

## 2. Material and methods

### 2.1. Reagents

[γ-^32^P] ATP was from PerkinElmer, MLi-2 was obtained from Merck [14]. Recombinant tetraubiquitin was generated by Dr Axel Knebel (MRC PPU). All mutagenesis was carried out using the QuikChange site-directed mutagenesis method (Stratagene) with KOD polymerase (Novagen). cDNA constructs for mammalian tissue culture (table 1) were amplified in *Escherichia coli* DH5*α* and purified using a NucleoBond Xtra Midi kit (no. 740 420.50; Macherey-Nagel). All DNA constructs were verified by DNA sequencing, which was performed by the Sequencing Service, School of Life Sciences, University of Dundee, using DYEnamic ET terminator chemistry (Amersham Biosciences) on Applied Biosystems automated DNA sequencers. DNA for bacterial protein expression was transformed into *E. coli* BL21 DE3 RIL (codon plus) cells (Stratagene). All cDNA plasmids (table 1), CRISPR gRNAs, antibodies and recombinant proteins generated for this study are available to request through our reagents website https://mrcppureagents.dundee.ac.uk/.

**Table 1.** List of cDNA constructs used with Dundee University (DU) identifier number.

DU number	expressed protein construct	variant
control constructs		
DU43407	PINK1-3FLAG (WT)	full-length WT
DU46669	PINK1-3FLAG D384A (KI)	full-length KI
DU46848	PINK1 (111–581)-3xFLAG	no MTS
DU51360	OPA3 (1-30)-PINK1 (111-581)-3xFLAG	OPA3-tether
truncation 111–130		
DU66367	HSP60 (1–27)-PINK1 104–581-3xFLAG	104-End
DU51362	HSP60 (1–27)-PINK1 (111–581)-3xFLAG	111-End
DU51855	HSP60 (1–27)-PINK1 (115–581)-3xFLAG	115-End
DU51817	HSP60 1–27 PINK1 (120–581)-3xFLAG	120-End
DU51864	HSP60 (1–27) PINK1 (125–581)-3xFLAG	125-End
DU51812	HSP60 (1–27) PINK1 (130–581)-3xFLAG	130-End
truncation 111–115		
DU66370	HSP60 (1–27)-PINK1 (112–581)-3xFLAG	112-End
DU66368	HSP60 (1–27)-PINK1 (113–581)-3xFLAG	113-End
DU66382	HSP60 (1–27)-PINK1 (114–581)-3xFLAG	114-End
A mutations		
DU66366	HSP60 (1–27)-PINK1 111-end-3xFLAG [I111A]	I-A
DU66363	HSP60 (1–27)-PINK1 111-end-3xFLAG [E112A]	E-A
DU66381	HSP60 (1–27)-PINK1 111-end-3xFLAG [E113A]	E-A
DU66359	HSP60 (1–27)-PINK1 111-end-3xFLAG [K114A]	K-A
DU66360	HSP60 (1–27)-PINK1 111-end-3xFLAG [Q115A]	Q-A
DU66369	HSP60 (1–27)-PINK1 111-end-3xFLAG [E117A]	E-A
DU66361	HSP60 (1–27)-PINK1 111-end-3xFLAG [E112A/E113A]	2E-A
DU66373	HSP60 (1–27)-PINK1–111–581–3xFLAG [E112A/E113A/E117A]	3E-A
E-K mutations		
DU66362	HSP60 (1–27)-PINK1 111-end-3xFLAG [E112K]	E-K
DU66365	HSP60 (1–27)-PINK1 111-end-3xFLAG [E113K)	E-K
DU66528	HSP60 (1–27)-PINK1 111-end-3xFLAG [E117K]	E-K
DU66364	HSP60 (1–27)-PINK1 111-end-3xFLAG [E112 K/E113K]	2E-K
DU66522	HSP60 (1–27)-PINK1 111-end-3xFLAG [E112 K/E113 K/E117K]	3E-K
PD mutations		
DU66374	HSP60-(1–27)-PINK1-(111–581)-3xFLAG (I111S)	I-S
DU66521	HSP60-(1–27)-PINK1-(111–581)-3xFLAG (Q115L)	Q-L
DU66376	HSP60-(1–27)-PINK1-(111–581)-3xFLAG (Q126P)	Q-P
DU66375	HSP60-(1–27)-PINK1-(111–581)-3xFLAG (C125G)	C-G
DU56048	pcDNA5-FRT/TO Pink1-3FLAG C125G	C-G
DU56186	pcDNA5-FRT/TO Pink1-3FLAG Q126P	Q-P
DU56077	pcDNA5-FRT/TO Pink1-3FLAG A168P	A-P
DU56079	pcDNA5-FRT/TO Pink1-3FLAG E240K	E-K
DU56049	pcDNA5-FRT/TO Pink1-3FLAG G309D	G-D
DU56085	pcDNA5-FRT/TO Pink1-3FLAG G409V	G-V
DU56537	pcDNA5-FRT/TO Pink1-3FLAG L539F	L-F
DU67934	pcDNA5-FRT TO PINK1 534_535InsQ 3FLAG	534_535insQ
TOM 20 tethering MTS		
DU66429	pOTC-(1–33)-PINK1-(111–581)-3xFLAG	WT MTS
DU66430	pOTC-(1–33)-PINK1-(111–581)-3xFLAG (L5A, L8A, L9A)	unable to bind TOM20
DU66451	F1 Beta-ATPase-(1–33)-PINK1-(111–581)-3xFLAG	WT MTS
DU66431	F1 Beta-ATPase-(1–33)-PINK1-(111–581)-3xFLAG (W29A, C32A, M33A)	unable to bind TOM20
DU66540	PINK1-M1-P34, I111-END-3xFLAG	35–110 deletion
CTE mutants		
DU27429	PINK1-3FLAG pcDNA5-FRT/TO (V528A)	V-A
DU60932	PINK1-3FLAG pcDNA5-FRT/TO (L532A)	L-A
DU60929	PINK1-3FLAG pcDNA5-FRT/TO (L539A)	L-A
DU67239	PINK1-3FLAG pcDNA5-FRT/TO (V528A/L532A/L539A)	3A
pediculus PINK1 mutations		
DU66324	PhPINK1 (108-end)	
DU66537	PhPINK1 (108-end) L507A	L-A
DU66538	PhPINK1 (108-end) L514F	L-F
DU66536	PhPINK1 (108-end) I503A	I-A
DU72026	PhPINK1 (108-end) W129P	W-P
DU66535	PhPINK1 (108-end) C128G	C-G
DU72025	PhPINK1 (108-end) E125K	E-K

### 2.2. Cell culture and transfection

SK-OV-3 and HeLa wild-type (WT) and PINK1 knockout cells were routinely cultured in standard DMEM (Dulbecco's modified Eagle's medium) supplemented with 10% FBS (fetal bovine serum), 2 mM L-Glutamine, 100 U ml^−1^ Penicillin, 100 mg ml^−1^ Streptomycin (1X Pen/Strep) and 1 X non-essential amino acids (Life Technologies). Flp-In T-REx HEK293 cells & HeLa cells were supplemented with 15 µg ml^−1^ of Blasticidin and 100 µg ml^−1^ of Hygromycin and were induced to express protein by addition of 0.1 µg ml^−1^ of Doxycycline to the medium for 24 h. All cells cultured at 37°C, 5% CO_2_ in a humidified incubator and routinely tested for Mycoplasma.

For transient expression, SK-OV-3 cells were transfected at 90% confluency using Lipofectamine 3000 (3.2 µl Lipofectamine and 4 µl P3000 reagent used per 10 cm plate). Cells were then cultured for a further 72 h including treatment of Oligomycin and Antimycin A for 9 h. HeLa cells were transiently transfected with 4–5 µg of DNA dissolved in 2 ml serum-free DMEM, mixed with 60 μl of 1 mg ml^−1^ PEI, vortexed gently for 10 s and incubated for 20–40 min at room temperature before being added dropwise to cells at around 50–60% confluency. To uncouple mitochondria, cells were treated with or without 10 µM CCCP (Sigma) dissolved in DMSO for 6 h or 10 µM Antimycin A/1 µM Oligomycin for 3 h. Cells were harvested in ice-cold lysis buffer as explained below.

### 2.3. Generation of CRISPR-Cas9 PINK1 knockout HEK293 cells and HeLa cells

CRISPR was performed using a paired Nickase approach to minimize off-target effects. Exon 2-specific guide pairs with low combined off-targeting scores were identified using the Sanger Institute CRISPR webtool (https://wge.stemcell.sanger.ac.uk/find_crisprs). Complementary oligos for the optimal guide pair A (G1 5′-gCTTGCAGGGCTTTCGGCTGG and G2 5′-gCGTCTCGTGTCCAACGGGTC) were designed and annealed according to the Zhang method with BbsI compatible overhangs facilitating cloning into the target vectors; the sense guide G1 was cloned into the puromycin selectable plasmid pBABEDPU6 (DU48788 https://mrcppureagents.dundee.ac.uk/) and antisense guide G2 cloned into the spCas9 D10A-expressing vector pX335 (Addgene Plasmid no. 42335) yielding clones DU52528 and DU52530, respectively. CRISPR was performed by co-transfection of Flp-In T-REx HEK293 cells/ Flp-In T-REx HeLa cells (60% confluency, 10 cm dish) with 3.75 µg of each plasmid in 27 µl Lipofectamine LTX according to manufacturer's instructions. Following CRISPR, media was removed and transfected cells were selected by twice incubation for 48 h in complete media supplemented with 3 µg ml^−1^ puromycin. Following the second incubation, cells were returned to fresh media lacking puromycin for recovery of selected cells. Single cells were isolated by flow-assisted cell-sorting into individual wells of a 96-well plate containing DMEM supplemented with 10% FBS, 2 mM L-glutamine, 100 U ml^−1^ penicillin, 100 µg ml^−1^ streptomycin and 100 µg ml^−1^ normocin (InvivoGen). After reaching approximately 80% confluency, individual clones were transferred into 6-well plates and PINK1 expression determined by immunoblot analysis following CCCP treatment. Knockouts were confirmed by sequencing.

Validated PINK1-knockout Flp-In T-REx HEK293clone ‘F1’ and Flp-In T-REx HeLa clone ‘3C9’ were used as the parental lines for the generation of Doxycycline-inducible, PINK1-3FLAG stably expressing cells. Cell lines were generated according to manufacturer's instructions by selection with hygromycin. Expression was confirmed by immunoblot following treatment with 20 ng ml^−1^ Doxycycline.

### 2.4. Generation of Flp-In T-REx PINK1-re-expression stable cell lines

To ensure low-level uniform expression of recombinant proteins, manufacturer's instructions (Invitrogen) were followed to generate stable cell lines that re-express C-terminal 3FLAG-tagged forms of PINK1 proteins (cDNA subcloned into pcDNA5-FRT/TO plasmid) in a doxycycline-inducible manner. Flp-In T-REx-293/Flp-In T-rex-Hela CRISPR knockout to PINK1 null cells were generated in a laboratory. The PINK1 null host cells containing integrated FRT recombination site sequences and Tet repressor were co-transfected with 4.5/9 µg of pOG44 plasmid (which constitutively expresses the Flp recombinase) and 0.5/1 µg of pcDNA5-FRT/TO vector containing a hygromycin resistance gene for selection of the gene of interest with FLAG tag under the control of a doxycycline-regulated promoter. Cells were selected for hygromycin and blasticidin resistance 3 days after transfection by adding fresh medium supplemented with 15 µg ml^−1^ of blasticidin and 100 µg ml^−1^ of hygromycin. Expression of the recombinant protein was induced by the addition of 0.1 µg ml^−1^ of Doxycycline for 24 h.

### 2.5. Primary human skin fibroblasts

Primary skin fibroblasts at low passage numbers (2–5) were contributed by the Hertie Institute for Clinical Brain Research Biobank, Tübingen, Germany. They were obtained from skin biopsies from a patient with PD with a PINK1 p.Q126P homozygous mutation (p.Q126P hom; c.388-7A > G hom; c.960-5G > A hom; c.*37A > T hom) [15] and age-matched healthy individuals following routine clinical procedures, underwritten informed consent (Hertie Institute for Clinical Brain Research Biobank) and approval by a local ethics committee (University of Tübingen). Patients were screened for GBA, LRRK2, PARK2, DJ-1 and PINK1 mutations by RFLP, MPLPA and bidirectional Sanger sequencing of the entire coding sequence of PINK1 using an ABI PRISM 3100-Avant Genetic Analyzer (Applied Biosystems), as described previously [15]. Fibroblasts were cultured in Dulbecco's modified Eagle's medium supplemented with glucose (4.5 g l^−1^), L-glutamine (2 mM), HEPES (10 mM), fetal bovine serum (10%) and penicillin (50 U ml^−1^)/streptomycin (50 lg ml^−1^) plus 1% (v/v) non-essential amino acid and grown at 37°C in a 5% CO_2_ atmosphere.

### 2.6. Whole-cell lysate preparation

Cells were lysed in an ice-cold lysis buffer containing 50 mM Tris-HCl, pH 7.45, 1% (by vol) Triton X-100, 250 mM sucrose, 1 mM EDTA, 1 mM EGTA, 1 mM sodium orthovanadate, 50 mM NaF, 10 mM 2-glycerophosphate, 5 mM sodium pyrophosphate and complete EDTA-free protease inhibitor cocktail (Roche) with freshly added 1x phosphatase inhibitor cocktail (Sigma-Aldrich). Lysates were clarified by centrifugation at 16 600*g* at 4°C for 20 min, and supernatants were quantified by BCA assay.

### 2.7. Isolation of mitochondrial-enriched fraction

Cells were collected in ice-cold mitochondria fractionation buffer containing 20 mM HEPES pH 7.5, 3 mM EDTA, 5 mM sodium β-glycerophosphate, 50 mM sodium fluoride, 5 mM sodium pyrophosphate, 250 mM sucrose, 1 mM sodium orthovanadate, 1x protease inhibitor cocktail (Roche) and 200 mM chloroacetamide. Cell pellets were disrupted by 25 passes in a handheld homogenizer, or 25-gauge needle, and lysates clarified by centrifugation at 800*g*, 10 min at 4°C. Supernatant was isolated and centrifuged at 16 000*g* for 10 min at 4°C. The resultant supernatant was retained as cytoplasmic fraction and the pellet was resuspended in mitochondria fractionation buffer with 1% Triton X-100 and retained as mitochondrial-enriched fraction. Samples were quantified by Bradford and normalized appropriately.

### 2.8. Blue native PAGE (BN-PAGE)

The samples for blue native PAGE (BN-PAGE) analysis were prepared using a Native PAGE Sample Prep Kit (Invitrogen). For BN-PAGE, mitochondria-enriched fractions were gently pipetted up and down 10 times in 1 × Native PAGE buffer with 1% digitonin followed by an incubation for 30 min at 4°C. The samples were centrifuged at 20 000*g* for 30 min at 4°C. Samples were quantified by BCA assay and supplemented with 0.002% G-250 (Invitrogen). BN-PAGE was performed by Native PAGE Running Buffers (Invitrogen). The gels were washed in denaturation buffer (10 mM Tris-HCl pH 6.8, 1% SDS and 50 mM DTT) for 15 min at 60°C after electrophoresis and then transferred on to PVDF membranes for IB analysis. PVDF membranes were incubated in destaining buffer (40% methanol, 10% acetic acid) and subjected to immunoblotting.

### 2.9. PINK1 immunoprecipitation

For immunoprecipitation of endogenous PINK1, 500 µg of whole cell was incubated overnight with PINK1 antibody (S085D, MRC PPU reagents and Services) coupled to Protein A/G beads as previously reported [16]. The immunoprecipitants were washed three times with lysis buffer containing 150 mM NaCl and eluted by resuspending in 10 µl of 2 × LDS sample buffer and incubating at 37°C for 15 min under constant shaking (2000 r.p.m.) followed by the addition of 2.5% (by vol) 2-mercaptoethanol.

### 2.10. Ubiquitin pulldown

For ubiquitylated protein capture, Halo-tagged ubiquitin-binding domains (UBDs) of TUBE (tandem-repeated ubiquitin-binding entities) was incubated with HaloLink resin (200 µl, Promega) in binding buffer (50 mm Tris HCl, pH 7.5, 150 mm NaCl, 0.05 % NP-40) overnight at 4°C. Membrane-enriched fraction (400 µg) was used for pulldown with HALO-UBDs. Halo Tube beads (20 µl) were added to whole-cell lysates for enrichment and incubated O/N at 4°C. The beads were washed three times with lysis buffer containing 0.25 mM NaCl and eluted by resuspension in 1 × LDS sample buffer (20 µl) with 1 mM dithiothreitol (DTT) or 2.5% 2-mercaptoethanol. The method for ubiquitin capture used has previously been reported for Halo-tagged UBDs of TUBE (tandem-repeated ubiquitin-binding entities) [17].

### 2.11. Immunoblotting

Samples were subjected to SDS-PAGE and transferred onto nitrocellulose membranes. Membranes were blocked with 5% BSA in TBS-T and incubated at 4°C overnight with the indicated antibodies, diluted in 5% BSA. Membranes for kinase assay screen were incubated with HRP-conjugated secondary antibodies (1 : 10 000) in 5% milk for 1 h at room temperature. Membranes were exposed with ECL substrate. Additional kinase assay immunoblotting membranes were incubated with secondary antibodies, conjugated with LICOR IRDye in TBS-T (1 : 10 000) and imaged using the LICOR Odyssey software.

### 2.12. Antibodies

The following primary antibodies were used: Parkin phospho-Ser65 rabbit monoclonal antibody was raised by Epitomics/Abcam in collaboration with the Michael J. Fox Foundation for Research (please contact tools@michaeljfox.org for questions). Ubiquitin phospho-Ser65 (Cell Signalling Technology (CST)), OPA1 (CST), PINK1 (Novus), PINK1 (in-house generated by Dundee Cell Products (DCP)), Parkin (Santa Cruz), Rab8A (Abcam), Rab8A phospho-Ser111 (Abcam), GAPDH (Santa Cruz) and Vinculin (CST). The polyclonal phospho-Ser228 PINK1 was generated by the Michael J. Fox Foundation's research tools program in partnership with Abcam (development of a monoclonal antibody is under way; please contact tools@michaeljfox.org for questions). The following antibody was produced by the MRCPPU Reagents and Services at the University of Dundee in sheep: anti-PINK1 (S085D).

### 2.13. Expression of recombinant pediculus PINK1 proteins

WT and mutant *Pediculus humanus corporis* PINK1 (Phc) (residues 108-end) were expressed as a His-SUMO tagged protein with SENP1 cleavage site in BL21(DE3)pLysS. Cells were grown in Terrific broth medium containing 50 µg ampicillin to an OD of 0.8 and expression was induced at 16°C with 250 µM IPTG and allowed to grow for a further 16 h. Cells were harvested by centrifugation at 4000 r.p.m. for 30 min, cell pellet was resuspended in lysis buffer; 25 mM Tris pH 8.5, 300 mM NaCl, 0.5 mM TCEP and 5% glycerol (containing lysozyme, DNAase and protease inhibitor cocktail and 10 mM imidazole). Cells were lysed by sonication and cell lysate centrifuged using ultra-centrifuge at 20 000*g* to collect cell debris. The supernatant was incubated with nickel beads at 4°C for 2 h on a rotatory platform. After 2 h, beads were collected and washed using lysis buffer containing 10 mM imidazole. Protein was eluted using lysis buffer containing 200 mM imidazole. His-SUMO was cleaved off overnight by dialysing into dialysis buffer (25 mM Tris, 300 mM NaCl pH 8.5, 0.5 mM TCEP, 5% glycerol) using His-SENP1 at 4°C. Cleaved protein was passed through fresh nickel beads to remove His-SUMO and His-SENP1. Eluted protein was collected, concentrated and purified further by gel filtration using gel filtration buffer (25 mM Tris, pH8.5, 150 mM NaCl, 5% glycerol and 0.5 mM TCEP). The elution profile of the protein was monitored using a calibrated Superdex 75 column. Pure fractions from SDS-PAGE analysis were collected, concentrated, quantified and flash frozen until needed.

### 2.14. Pediculus PINK1 *in vitro* kinase assay

*In vitro* activity assays were set up in a final volume of 40 µl containing 2 µM substrate (K63-linked Tetra-Ub) in 50 mM Tris-HCl pH 7.5, 0.1 mM EGTA, 10 mM MgCl2 and 0.1 mM [γ-32P]ATP (approximately 500 cpm pmol^−1^), and 250 nM of PhcPINK1 (WT) and mutants as kinase were used. Assays were incubated at 30°C for 10 min with shaking at 1050 rpm and terminated by the addition of 13 µl 4 × LDS sample buffer containing a reducing agent. Samples were boiled at 70°C for 10 min and reactions resolved by SDS-PAGE. For autoradiography, gels were stained for protein detection for 1 h in Coomassie InstantBlue and then destained by washes in warm water. Wet gels were scanned in an Epson scanner, then dried completely using a gel dryer (Bio-Rad). Incorporation of [*^γ^*^32^P]ATP into substrates was analysed by exposure to Amersham Hyper-Film at −80°C. For assay quantification, individual SDS-PAGE bands were excised from dried gels and incorporation determined by Cerenkov counting. Kinase activity is determined by quantification of the number of phosphates transferred by a kinase, per minute of the reaction, per kinase molecules in the reaction. Number of phosphates transferred is determined by relating the scintillation counts of a substrate band to the counts of a known amount of phosphate (i.e. the 1 mM [^y−32^P]ATP stock).

### 2.15. Intact mitochondrial kinase assay

Intact mitochondrial assay was performed as previously described [18]. Intact mitochondria-enriched fractions were isolated in cell-free assay buffer (CFAB: 20 mM HEPES KOH (pH 7.5), 220 mM sorbitol, 10 mM potassium acetate, 70 mM sucrose protease inhibitor cocktail minus EDTA (Roche)) and were incubated with 2 µM substrate (tetraubiquitin (K63 linked)) 2 mM ATP, 2 mM DTT, 5 mM MgCl2 and 1% glycerol (50 µl final) for indicated time points. Reactions stopped by addition of 16.6 µl 4 x LDS 4% 2-beta-mercaptoethanol and samples were vortexed and heated at 95°C for 5 min. Samples were diluted 1 : 10 in 1 × LDS for substrate immunoblotting and remainder used for immunoblotting controls and Coomassie staining.

### 2.16. Hydrogen/deuterium exchange mass spectrometry

The hydrogen/deuterium exchange mass spectrometry (HDX) experiment was performed as described by Cornwell *et al.* [19] with modifications. Briefly, 5 µl of 5 µM of WT and L507A mutant PhcPINK1 (residues 108–575) were equilibrated in 50 mM potassium phosphate, 300 mM sodium chloride, 5 mM TCEP, pH 7.2. Samples were mixed with 95 µl deuterated buffer (10 mM potassium phosphate, 300 mM sodium chloride and 5 mM TCEP pD 6.8) and were then incubated at 4°C for the specified times (0.5 min or 2 min). At the end of the labelling time, 100 µl of the reaction mixture was added to 100 µl of the quench buffer at 0°C (10 mM potassium phosphate, 5 mM TCEP, 0.1% DDM. The quench buffer pH was adjusted so the final mixture was at pH 2.5). Fifty microlitres of the quenched sample was passed through an immobilized pepsin column (Affipro, Prague, Czech Republic) at 150 µl min^−1^ at 20°C, and the resulting peptides were trapped by a VanGuard Acquity UPLC BEH C18 pre-column for 3 min. The peptides were then transferred to a C18 column (75 µm × 150 mm, Waters Ltd., Wilmslow, Manchester, UK) and separated by gradient elution of 5–40% MeCN in water and 0.1% formic acid over 7 min at 40 µl min^−1^. Trapping and gradient elution of peptides was performed at 0°C.

The HDX system was interfaced to a Synapt G2Si mass spectrometer (Waters Ltd., Wilmslow, Manchester, UK). HDMS^E^ and dynamic range extension modes (data-independent analysis coupled with IMS separation) were used to separate peptides prior to CID fragmentation in the transfer cell [20]. Data were analysed using PLGS (v. 3.0.2) and DynamX (v. 3.0.0) software, which were supplied with the mass spectrometer as previously described [19].

## 3. Results

### 3.1. Mitochondrial import and association via the TOM complex is required for PINK1 activation

**Figure 1.**
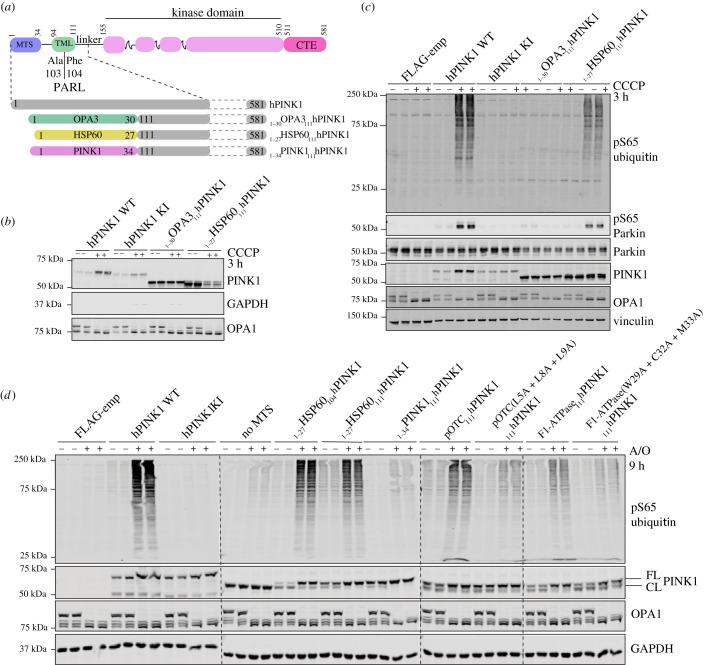
MTS that mediate TOM complex association are required for PINK1 activation. (*a*) Schematic depiction of constructs for human PINK1 (hPINK1) mutants. For hPINK1 mutants targeted to different compartments of the mitochondria, residues 1–110, which includes the MTS and transmembrane-like domain (TML) and PARL cleavage site of hPINK1, were removed and replaced with the predicted MTS of proteins known to localize to the OMM (OPA3 1–30) and matrix (HSP60 1–27). For hPINK1 mutant with predicted MTS of PINK1, residues 1–110 were replaced with hPINK 1 residues 1–34. (*b*) Flp-In TRex HEK293 hPINK1 KO cells stably expressing WT, KI (D384A), OPA3 (1–30)-hPINK1(111–581) or HSP60 (1–27) hPINK1(111–581) were treated ±10 µM CCCP for 3 h and subjected to sub-cellular fractionation to obtain membrane-enriched fraction. Samples were resolved by SDS-PAGE. Proteins were transferred to nitrocellulose membranes probed using the antibodies indicated. *n* = 2. (*c*) Flp-In TRex HEK293 cells PINK1 KO stably expressing empty vector (FLAG-emp), hPINK1-3FLAG WT, kinase inactive (KI, D384A), OPA3 (1–30)-hPINK1 (111–581) and HSP60 (1–27)-hPINK1 (111–581) were treated with ±10 µM CCCP for 3 h. Whole-cell lysates (Parkin and control blots) or membrane-enriched fractions (FLAG and pSer^65^ ubiquitin blots) were resolved by SDS-PAGE. Proteins were transferred to nitrocellulose membranes probed using the antibodies indicated. (*d*) Human SK-OV-3 PINK1 knockout cells were transfected with empty vector (FLAG-emp), hPINK1-3FLAG WTkinase inactive (KI, D384A), HSP60 (1–27)-hPINK1 (104–581), HSP60 (1–27)-hPINK1 (111–581), hPINK1 (1–34)-hPINK1 (111–581), pOTC (1–30)-hPINK1 (111–581) and pF1ATPase (1–30)-hPINK1 (111–581). Cells were treated with A/O for 9 h prior to lysis and probed using the indicated antibodies.

It has previously been reported that stable expression of PINK1 at the OMM using a fusion construct of N-terminally truncated PINK1 with the OMM anchor sequence of OPA3 (OPA3 (1–30)-PINK1 (111-end) is sufficient to activate human PINK1 independent of mitochondrial depolarization as determined using the Parkin-mitochondria recruitment assay [21]. We initially explored whether PINK1 stabilization at the OMM or association with the TOM complex is sufficient for its activation as measured by monitoring phosphorylation of its substrates Parkin and ubiquitin. We first generated Flp-In T-REx HEK293 PINK1-knockout cells by exon 2-targeted CRISPR-Cas9 (electronic supplementary material, figures S1A,B), then stably re-introduced full-length PINK1-3FLAG WT; kinase-inactive mutant (KI; D384A); OPA3 (1–30)-PINK1_111-581_ (OPA3-_111_PINK1); or PINK1 fused to a conventional N-terminal MTS that is imported to the matrix via the TOM, namely HSP60 (1–27)-PINK1_111-581_ (HSP60-_111_PINK1) (figure 1*a*). Cells were treated with DMSO or 10 µM CCCP for 3 h, and following cell lysis, mitochondrial extracts were analysed by immunoblotting with anti-FLAG antibody (figure 1*b*). This confirmed that HSP60-_111_PINK1 underwent proteolytic cleavage under basal conditions indicating successful import to the mitochondrial matrix, and this was reduced upon mitochondrial depolarization (figure 1*b*). By contrast, OPA3-_111_PINK1 was stably expressed at the mitochondria with no evidence of proteolysis under basal conditions and no change upon mitochondrial depolarization, suggesting that it is stabilized at the OMM and not imported (figure 1*b*). Cells were next transfected with untagged Parkin and treated with DMSO or CCCP for 3 h. Cells were lysed and whole-cell extracts analysed by immunoblotting with anti-phospho-Ser65 ubiquitin and anti-phospho-Ser65-Parkin antibodies (figure 1*c*). Under basal conditions, we observed robust stable levels of OPA3-_111_PINK1; however, this was not active under our assay conditions and furthermore we did not observe any activation of OPA3-_111_PINK1 following mitochondrial depolarization (figure 1*b*). By contrast, we observed that HSP60-_111_PINK1 was robustly activated upon mitochondrial depolarization with induction of Ser65-phosphorylated-ubiquitin (phospho-ubiquitin) and Ser65-phosphorylated-Parkin (phospho-Parkin) similar to WT PINK1 (figure 1*c*).

To confirm these results, we used human SK-OV-3 ovarian cancer cells that we have identified as a robust system to study endogenous PINK1-Parkin signalling [22]. We performed transient expression of WT PINK1, KI PINK1 and HSP60-_111_PINK1 in SK-OV-03 PINK1-knockout cells followed by treatment with or without A/O for 9 h and observed induction of phospho-ubiquitin in cells expressing HSP60-_111_PINK1 upon mitochondrial depolarization consistent with results in Flp-In T-REx HEK293 cells (figure 1*d*). The activation of PINK1 was critically dependent on MTS-directed import since the expression of _111_PINK1 without a MTS was not activatable (figure 1*d*). Furthermore, we did not observe any difference in activation between HSP60-_104_PINK1 and HSP60-_111_PINK1. We next tested the generality of our findings using two well-studied MTS sequences, pOTC (1–30) and pF1-β ATPase (1–30), which have been shown to mediate mitochondrial import via TOM association [23]. Upon mitochondrial depolarization, we observed that both pOTC-_111_PINK1 and pF1-β ATPase-_111_PINK1 led to activation and induction of phospho-ubiquitin. By contrast, mutant sequences of pOTC (L5A/L8A/L9A) and pF1-β ATPase (W29A/C32A/M33A) that have been shown to impair TOM complex interaction [23] were associated with significantly reduced PINK1 activation and phospho-ubiquitin following mitochondrial depolarization (figure 1*d*). We have previously shown that the PINK1 MTS (residues 1–34) is sufficient to localize PINK1 to mitochondria [24]. Interestingly, we observed that PINK1[1–34]-_111_PINK1 was imported and cleaved under basal conditions; however, we did not observe any activation following mitochondrial depolarization suggesting that additional residues within its N-terminus are required to stabilize it within the TOM complex for activation (figure 1*d*). This is consistent with previous studies that have suggested that the sequence determinants for PINK1 import in the native protein are complex, redundant and may include additional MTS sequences located between residues 35–90 [25], and in future studies, it will be interesting to define those sites required for activation.

### 3.2. Mapping the minimum boundary of PINK1 required for activation in cells

**Figure 2.**
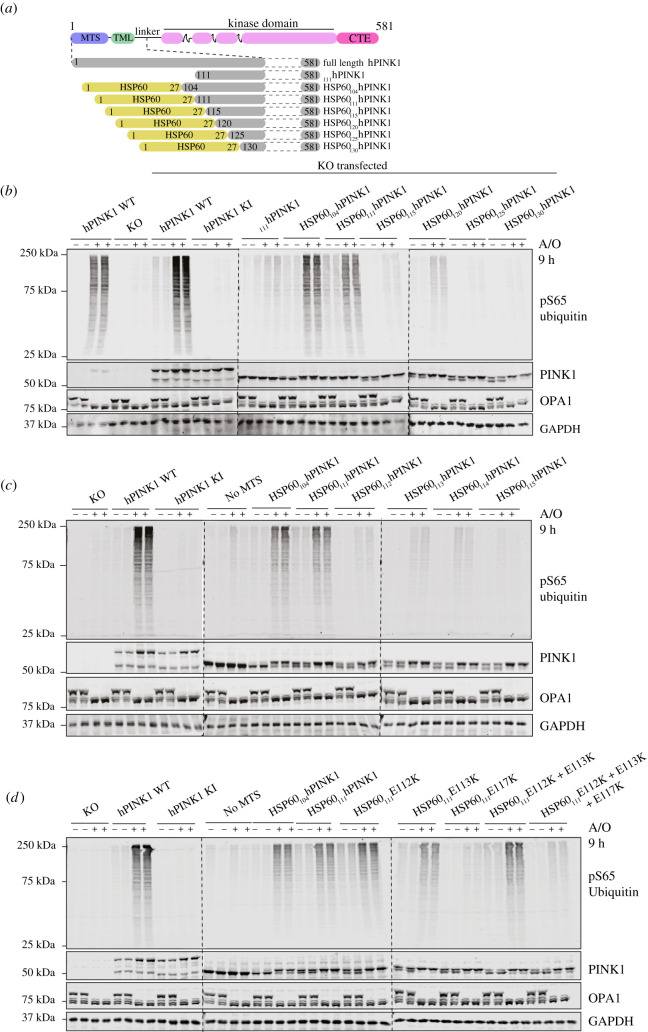
Mapping of N-terminal boundary of minimal region required for PINK1 activation to residue Ile111. (*a*) Schematic depiction of deletion mutants of human PINK1 (hPINK1) (start sites varying from 104–130 to END) fused at the N-terminus with HSP60-MTS aa 1–27. (*b*) Cell-based analysis of WT and hPINK1 knockout (PINK1 KO) SK-OV-3 cells transfected with indicated deletion mutants of hPINK1 ranging from HSP60(1–27)-hPINK1(104–581) to HSP60(1–27)- hPINK1(130–581) alongside full-length WT and kinase inactive (KI) hPINK1. Cells were treated with A/O for 9 h prior to lysis and immunoblotted with indicated antibodies (anti-pSer65 ubiquitin, anti-PINK1, anti-OPA1 and anti-GAPDH). The membranes were developed using the LI-COR Odyssey CLx Western blot imaging system. (*c*) Cell-based analysis of PINK1 KO SK-OV-3 cells transfected with deletion mutants of hPINK1 ranging from HSP60(1–27)-hPINK1(111–581) to HSP60(1–27)-hPINK1(115–581). Cells were treated with A/O for 9 h prior to lysis and immunoblotting with indicated antibodies and analysed as described above. (*d*) Cell-based analysis of PINK1 KO SKOV3 cells transfected with N-terminal hPINK1 E-K single mutants (E112K or E113K or E117K), double mutant (E112K + E113K) and triple mutants (E112K + E113K + E117K) of the region spanning residues 111–117 of HSP60(1–27)-hPINK1(111–581). Cells were treated with A/O for 9 h prior to lysis, blotted with indicated antibodies and analysed as described above.

To better define the regions of human PINK1 required for optimal activation and phosphorylation of ubiquitin, we initially expressed a series of PINK1 constructs in SK-OV-3 PINK1 KO cells including HSP60-_130_PINK1, HSP60-_125_PINK1, HSP60-_120_PINK1, HSP60-_115_PINK1, HSP60-_111_PINK1 and HSP60-_104_PINK1 (figure 2*a*). Immunoblotting analysis with anti-PINK1 antibodies indicated that all PINK1 constructs were expressed at similar levels; however, upon A/O treatment, we only observed robust activation of PINK1 in cells expressing either HSP60-_104_PINK1 or HSP60-_111_PINK1 that paralleled WT PINK1 and endogenous PINK1 activation (figure 2*b*). Since HSP60-_115_PINK1 was not sufficient for PINK1 activation, we performed fine mapping of the region between residue Ile111 and Gln115 and expressed a series of further constructs including HSP60-_114_PINK1, HSP60-_113_PINK1, HSP60-_112_PINK1 and HSP60-_111_PINK1. All constructs were expressed at similar levels and underwent cleavage and processing under basal conditions followed by stabilization of the upper PINK1 band upon mitochondrial depolarization (figure 2*c*). However, there was a strict requirement for the N-terminal region of PINK1 to start at residue Ile111 (HSP60-_111_PINK1) for subsequent activation by mitochondrial depolarization (figure 2*c*). The region of PINK1 between Ile111 and the kinase domain, spanning residues 111–156, is colloquially referred to as the PINK1 ‘linker’ region, and no structural information for this region is currently published. A previous study found that triple mutation of a cluster of negatively charged glutamic acid residues (Glu112Ala/Glu113Ala/Glu117Ala) in this region disrupted accumulation of PINK1 in depolarized mitochondria and its subsequent activation and recruitment of Parkin [26]. We initially assessed the role of these amino acids in the activation of HSP60-PINK1_111-581_, by mutating each Glu residue to alanine; however, these did not significantly impact PINK1 activation (data not shown). We next substituted the charge state of each Glu to a Lys residue and expressed these variants in PINK1 KO SK-OV-3 cells. All constructs were expressed at similar levels and underwent cleavage and processing under basal conditions followed by stabilization of the upper band upon mitochondrial depolarization (figure 2*d*). However, the Glu117Lys (E117 K) mutation was sufficient to completely prevent PINK1 activation, whereas there was no effect of mutation of the other Glu residues including the double Glu112 K/Glu113 K mutant (figure 2*d*).

### 3.3. Structural modelling predicts N-terminal α-helix extension domain upstream of kinase domain of PINK1 that interacts with CTE

**Figure 3.**
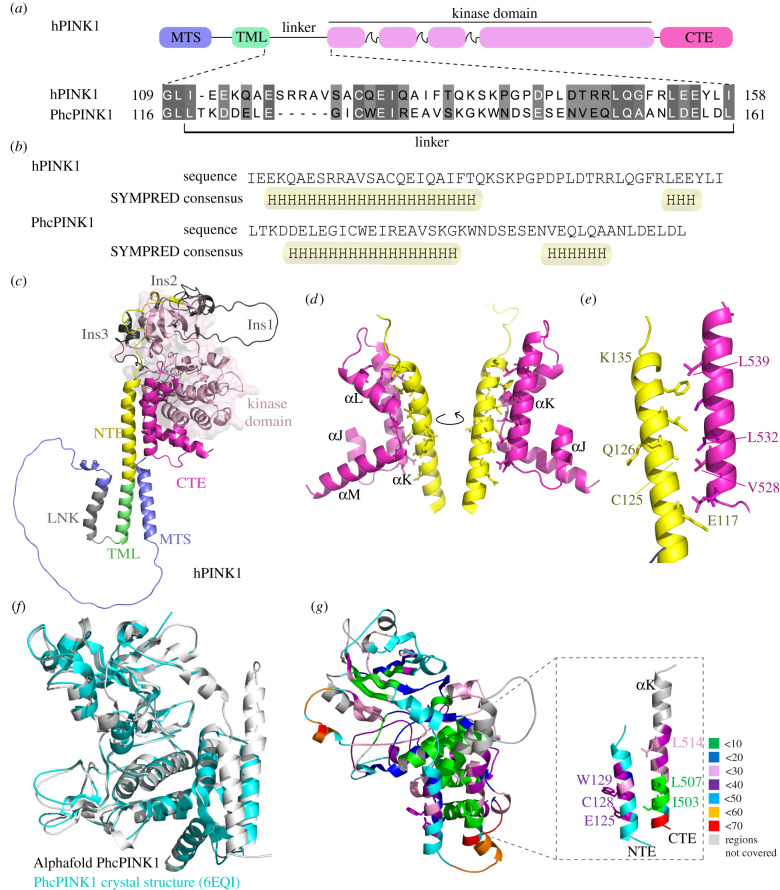
Structural modelling predicts an N-terminal α-helix extension (NTE domain) and its interaction with CTE domain. (*a*) Prediction of α-helix in the linker region of PINK1 flanked by transmembrane domain and kinase domain. Schematic representation of human PINK1 (hPINK1) and *Pediculus humanus corporis* PINK1 (PhcPINK1) highlighting the linker region flanked by transmembrane domain and kinase domain. The alignment of linker region is performed by MAFT and annotated in Jalview. (*b*) Prediction of secondary structure in linker region of hPINK1 and PhcPINK1 by SYMPRED. SYMPRED predicted α-helix represents a consensus prediction of α-helix by different prediction tools; PROF, SSPRO, YASPIN, JNET and PSIPRED. Predicted α-helices are highlighted by cylinders. (*c*) Complete structure of human PINK1 (hPINK1) solved by AlphaFold (Uniprot ID: Q9BXM7) and colour coded according to regions. (*e*) α-NTE domain of hPINK1 forms intramolecular interaction with CTE domain. α-NTE region primarily interacts with *α*K region of CTE. (*e*) Location of conserved residues at the NTE:CTE interface; PD-associated residues are highlighted. The residues of α-NTE at the start of interface (E117) and end of α-helix (K135) are also highlighted. (*f*) AlphaFold predicted PhcPINK1(108–575) model (grey) superposed with the crystal structure of PhcPINK1 (6EQI, blue) with a rmsd of 0.7. The missing NTE region in the crystal structure is labelled. (*g*) HDX data mapped on AlphaFold modelled PhcPINK1(108–575) with zoomed in view of NTE and CTE. The percentage deuterium uptake across the protein after 2 min of labelling is colour-coded as labelled. Residues also colour-coded according to the percentage deuterium uptake.

Current insect structures do not include the PINK1 ‘linker’ region spanning residues 111–156 (figure 3*a*) [9,10]. We therefore subjected the linker regions of human PINK1 and PhcPINK1 to secondary structure prediction using SYMPRED [27]. For human PINK1, secondary structure prediction tools consistently return an α-helical structure, reaching a consensus for residues Ile111 - Lys135 with a loop linking the α-helix to the kinase domain (figure 3*b*; electronic supplementary material, figure S2A,B). A similar consensus is reached for PhcPINK1 with tools predicting an α-helix from Lys120-Trp140 followed by a poorly defined region linking to the kinase domain (figure 3*b*; electronic supplementary material, figure S2A,C). Furthermore, we analysed the structural model of human PINK1 using AlphaFold [13], and this predicted with high confidence the presence of the NTE domain extending to Lys135 (figure 3*c–e*; electronic supplementary material, figures S2D). These regions are highly conserved within the overall PINK1 sequence (electronic supplementary material, figure S3).

Furthermore, an inspection of the human PINK1 AlphaFold model suggests that the N-terminal α-helix forms an intramolecular interface with the *α*K helix of the CTE (figure 3*c*–*e*). Of relevance is the location of residues mutated in PD including Cys125 and Gln126 within this interface as well as Glu117. The key residues in the *α*K helix mediating the interaction include Val528, Leu532 and Leu539 (figure 3*c*–*e*) in which a PD case has been reported with homozygous Leu539Phe mutation [28].

The AlphaFold human PINK1 structure model and PhcPINK1 crystal structure resemble structural arrangements with the NFK3 pseudokinase family members, PEAK1 and Pragmin that contain helical regions on either side of the pseudokinase domain, known as split helical dimerization (SHeD) (electronic supplementary material, figure S4A) [29,30]. The N-terminal region (SHeD1) forms a single 24-residue α-helix (termed αS) similar to the predicted NTE domain for human PINK1 and PhcPINK1 (electronic supplementary material, figure S4B). Most strikingly, the SHeD2 region of PEAK1 is highly similar to the PINK1 CTE domain with four α-helices (termed αJ-M) and superimposition of the PhcPINK1 CTE and PEAK1 SHeD2 reveals a high degree of structural similarity (electronic supplementary material, figures S4C,E). PEAK1 uses the surface of the αK and αL helices for a cis-contact with the SHeD1 αS-helix via ionic and hydrogen bond interactions (electronic supplementary material, figure S4F). This structurally resembles the AlphaFold predicted interface in PINK1 which is mediated by the arrangement of hydrophobic residues on the surface of the NTE domain and αK-helix and the conservation of these hydrophobic residues in PhcPINK1 suggests a similar interaction of the NTE domain with the CTE domain (electronic supplementary material, figure S4G).

We next expressed and purified an N-terminal extended fragment of PhcPINK1 (residues 108–575) containing the predicted NTE domain (electronic supplementary material, figure S5) and performed HDX mass spectrometry (HDX-MS) to gain molecular insights into the putative NTE domain of PINK. HDX-MS gives relative rate of exchange backbone amide hydrogen with deuterium based on the strength of hydrogen bonding and solvent accessibility in a well-ordered protein. It also can be used to distinguish peptides that are in a protein core (low uptake over time) from those that are exposed (high uptake) [31]. The HDX pepsin digestion yielded 185 peptides covering 85% of the entire sequence (electronic supplementary material, figure S6) and regions not covered were excluded from the analysis. Deuterium uptake after 2 min was used for the analysis as saturation was reached at this time. The rate of uptake was then colour coded and mapped on to an AlphaFold predicted structure of PhcPINK1 (residues 108–575) (figure 3*f,g*). Peptides with uptake of less than 60% are considered not to be a random coil but a well-structured region. Consistent with the AlphaFold predicted model, the HDX analysis demonstrated that peptides within the NTE domain-containing region of PhcPINK1 exhibited low-medium level of deuterium uptake indicating that the region is well ordered (figure 3*g*). We next performed HDX-MS analysis on a CTE domain, L507A, mutant of PhcPINK1 (orthologous to residue L532 of human PINK1) (electronic supplementary material, figure S7A,B). Comparative analysis of HDX-MS data between the WT PhcPINK1 and L507A mutant strikingly revealed significant differences in deuterium uptake localized within the NTE region (electronic supplementary material, figure S7C,D). Specifically we observed higher deuteration rates in two NTE-localized peptides spanning amino acid residues 125–129 and 125–130 of the L507A mutant compared to WT PhcPINK1 (electronic supplementary material, figures S7C,D). This suggests that the L507A mutation may specifically disrupt hydrophobic interaction between the CTE and residue Ile127 of the NTE leading to conformational changes and increased solvent accessibility of the NTE region (electronic supplementary material, figure S7A–D). Consistent with this we observed no significant difference in deuteration between L507A and WT PhcPINK1 peptides from regions outside the NTE including the N-lobe of the catalytic kinase domain (electronic supplementary material, figure S7E), C-lobe of the catalytic kinase domain (electronic supplementary material, figure S7F) and the C-terminus peptide spanning residues 571–575 (electronic supplementary material, figure S7G).

### 3.4. Biochemical analysis indicates critical role of NTE : CTE interface for PINK1 activation in cells

The location of PD-associated pathogenic mutations within the NTE domain suggests that the AlphaFold predicted NTE : CTE interaction is critical for PINK1 function. The generated model indicates that mutations in the conserved Cys125 residue (C125G) could disrupt the NTE : CTE interface while the Q126P mutation would break the NTE domain α-helix (figure 3*e*). To investigate the functional impact of PD mutations at the interface, we generated stable cell lines in which we re-introduced full-length PINK1-3FLAG WT; kinase-inactive mutant PINK1 (KI); NTE domain PINK1 mutants namely C125G, Q126P; CTE mutants PINK1 534_535InsQ and L539F, and kinase domain mutants namely A168P, E240 K, G309D and G409 V, into Flp-In T-REx HeLa PINK1-knockout cells (generated by exon 2-targeted CRISPR-Cas9 (electronic supplementary material, figure S8A,B)). Structural data from the insect orthologues indicate that A168P and E240 K mutations disrupt ATP binding while G309D lies within INS3 and disrupts substrate binding without affecting autophosphorylation of Ser228 [9,10]. G409 V lies on the P + 1 loop thereby preventing substrate recognition from the C-lobe side and has previously been reported to preserve autophosphorylation but disrupt downstream signalling [12].

**Figure 4.**
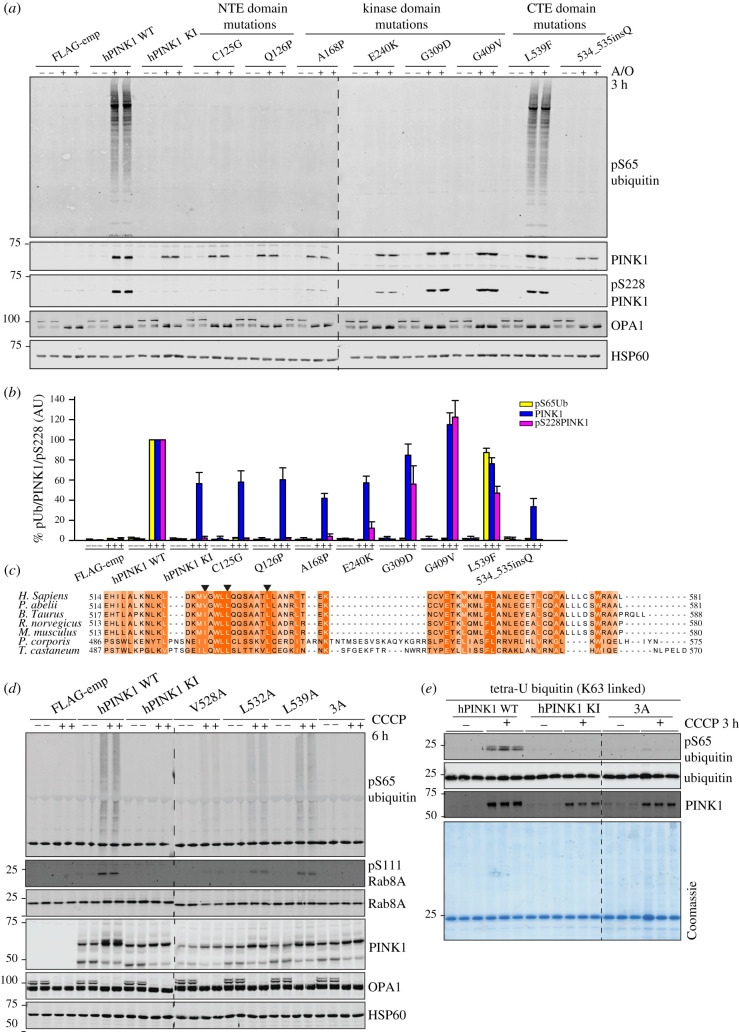
Mutational analysis confirms the critical role of NTE and CTE domains for PINK1 activation. (*a*) α-NTE and CTE PD-associated mutants lead to reduced protein stabilization, and loss of autophosphorylation and hPINK1 activation. Stably expressing PINK1-3FLAG WT, KI (D384A), empty vector (FLAG-emp), α-NTE mutants (C125G, Q126P), kinase domain mutants (A168P, E240K, G309D, G409V) and CTE domain mutants (L539F, ins534Q) cell lines were generated in PINK1-knockout Flp-In TRex-HeLa cells. PINK1-3FLAG expression was induced by 24 h treatment with 0.2 uM doxycycline, and mitochondrial depolarization induced by 3 h treatment with 10 µM A/O where indicated. Mitochondrial-enriched fractions were subjected to immunoblotting with α-PINK1 (in-house/DCP antibody), α-ubiquitin pS65 (CST), α-OPA1 (BD) and α-HSP-60 primary antibodies. *n* = 3. (*b*) Immunoblots were quantified for phospho-Ser65 Ub/HSP-60, PINK1/HSP-60 and pS228/HSP-60 using Image Studio software. Data are presented relative to WT hPINK as mean ± s.d. (*n* = 3). (*c*) Multiple sequence alignment of CTE region of PINK1 orthologues across species. Sequence alignment was performed with MUSCLE and annotated in Jalview. Mutated CTE residues for functional analysis are highlighted with arrow heads. (*d*) CTE mutants exhibit reduced stabilization, autophosphorylation and substrate phosphorylation. hPINK1 knockout HeLa cells transiently expressing hPINK1-3FLAG WT, KI or hPINK1 CTE mutants V528A (L503A in PhcPINK1), L532A (L507A in PhcPINK1) and L539A (L514A in PhcPINK1). Cells were stimulated ±10 μM CCCP for 6 h. Membrane fractions were isolated and solubilized in 1% Triton X-100 lysis buffer Lysates that were resolved by SDS-PAGE. Proteins were transferred to nitrocellulose membranes probed using the antibodies indicated. *n* = 2. (*e*) CTE triple mutant is inactive against recombinant substrates *in vitro*. Flp-In HEK293 PINK1 KO cells stably expressing WT or KI (D384A) or dimerization triple mutant (V528A/L532A/L539A) PINK1 were treated ±10 µM CCCP for 3 h and subjected to sub-cellular fraction in a CFAB. Three micrograms of non-solubilized membrane-enriched fraction (MeF) were incubated with tetraubiquitin (K63 linked) in CFAB supplemented with 2 mM (unlabelled) ATP, 5 mM MgCl2, 2 mM DTT and 0.75% Glycerol for 20 min. Samples were resolved via SDS-PAGE and blotted for the indicated antibodies. *n* = 2.

To determine the effect of the selected PINK1 mutants on activation, cells were treated with DMSO or A/O for 3 h to induce mitochondrial depolarization. Mitochondrial-enriched fractions were isolated and solubilized in 1% Triton-X100 lysis buffer. Immunoblot analysis of PINK1 demonstrated a significant reduction of PINK1 levels in mitochondria for the N-terminal mutants C125G, Q126P and the CTE mutant 534_535InsQ compared to WT PINK1 following mitochondrial depolarization (figure 4*a*,*b*). We also observed decreased PINK1 expression in the ATP-binding-defective mutants, A168P and E240K, but there was no alteration of expression for the substrate binding-defective mutants, G309D and G409 V (figure 4*a*,*b*). To monitor autophosphorylation, we employed a polyclonal phospho-specific Ser228 antibody [32] (see Material and Methods) and observed a complete loss of Ser228 autophosphorylation in the C125G, Q126P and 534_535InsQ mutants similar to PINK1 KI-expressing cells (figure 4*a*,*b*). We observed no reduction of Ser228 autophosphorylation in the substrate-binding mutants, G309D and G409V, which is consistent with previous studies of these mutants in insect PINK1 (G309D) and mammalian cells (G409V) [9,10,12,33]. Interestingly, we saw residual Ser228 autophosphorylation in the A168P and E240K mutant suggesting that these do not completely abolish catalytic activity (figure 4*a*,*b*). Finally C125G, Q126P, A168P, E240K, G309D, G409V and 534_535InsQ were associated with complete loss of phospho-ubiquitin upon mitochondrial depolarization, that would disrupt downstream signalling consistent with their pathogenic role in the development of PD (figure 4*a*,*b*).

Under these assay conditions, we did not observe any impact of the L539F mutation on PINK1 stabilization, autophosphorylation or ubiquitin phosphorylation (figure 4*a*,*b*). Based on the AlphaFold human PINK1 structure model, the NTE : CTE interface is facilitated by key hydrophobic residues on the surface of the *α*K-helix composed of Val528, Leu532 and Leu539 residues that are well-conserved across species (figures 3*e* and 4*c*). Since phenylalanine (Phe) is also hydrophobic, it would be anticipated that the L539F mutation would not be deleterious to the interface. To validate the functional role of the hydrophobic CTE interface, we transiently transfected WT and KI PINK1-3FLAG alongside CTE mutants, V528A, L532A, L539A and a combinatorial V528A/L532A/L539A triple mutant (3A) in Flp-In T-REx HeLa PINK1-knockout cells followed by 6 h treatment with DMSO or 10 µM CCCP. Immunoblot analysis of PINK1 in mitochondrial-enriched fractions demonstrated reduced PINK1 levels for all the CTE mutants, V528A, L532A and L539A, as well as in the 3A mutant compared to controls (figure 4*d*). Activation of PINK1 was assessed by immunoblotting against substrates, phospho-ubiquitin and phospho-Ser111-Rab8A (pSerRab8A) and all mutants led to reduced phospho-ubiquitin and pSerRab8A—most notable in the V528A, L532A mutants—and a lesser reduction in the L539A mutant (figure 4*d*). Strikingly, PINK1 activation was completely abolished in the 3A mutant (figure 4*d*). To further assess how the CTE mutants affect mitochondrial localization and activity of PINK1, we performed an *in vitro* intact MitoKA of PINK1 (electronic supplementary material, figure S9) [18]. We isolated mitochondria-enriched fractions from Flp-In T-REx HEK293 PINK1-knockout cells stably re-expressing WT, KI or the 3A mutant of human PINK1 and subjected these extracts to *in vitro* kinase assay using recombinant K63-linked tetraubiquitin (K63-Ub_4_) (figure 4*e*). Immunoblotting of PINK1 demonstrated decreased PINK1 levels in the isolated mitochondria of PINK1 KI and 3A-expressing cells following mitochondrial depolarization (figure 4*e*). Under these assay conditions, WT PINK1 that was localized to mitochondria efficiently phosphorylates K63-Ub_4_; however, this was completely abolished in mitochondria isolated from KI PINK1 and 3A PINK1-expressing cells (figure 4*e*). Overall these studies demonstrate the critical role of the CTE hydrophobic residues in PINK1 activation and strongly suggest that the L539F mutant is not pathogenic and causal of PD in the case reported [28].

The location of the NTE : CTE interface outside the kinase domain suggests that mutants would not directly affect the intrinsic catalytic activity of PINK1. Previous studies have found that the expression of recombinant human PINK1 in *E.* *coli* displays very low catalytic activity, whereas the expression of insect PINK1 orthologues exhibit robust activity *in vitro*. To determine the impact of NTE domain and CTE mutations on PINK1 catalytic activity, we performed an *in vitro* kinase using recombinant WT and mutant PhcPINK1 (residues 108–575) expressed in *E. coli*, incubated with [γ-^32^P]-ATP and K63-Ub_4_ substrate. Consistent with the AlphaFold structure prediction, we observed that all mutants tested, namely E125K, C128G, W129P, I503A, L507A and L514F (equivalent to human E117K, C125G, Q126P, V528A, L532A and L539F, respectively), efficiently phosphorylated K63-Ub4, similar to WT PINK1 as determined by measuring [γ-^32^P]-ATP incorporation via autoradiography (electronic supplementary material, figure S10A). To determine whether NTE and CTE domain mutants affect the ability of PINK1 to associate with the TOM complex, we generated mitochondrial-enriched fractions from HeLa PINK1 KO cell lines stably re-expressing WT, KI, S228A, C125G, Q126P and 534_535InsQ mutant PINK1, and subjected these to BN-PAGE assays. Consistent with previous studies, we observed that WT PINK1 stabilizes in an approximately 700 kDa complex in response to mitochondrial depolarization (electronic supplementary material, figure S10B). We observed that the C125G, Q126P and 534_535InsQ mutants had reduced PINK1 levels at the mitochondria similar to KI PINK1 and the S228A mutant, but there was a striking loss of association with the TOM complex to a greater degree than KI or S228A PINK1 (electronic supplementary material, figure S10B). This suggests that a major role of the NTE : CTE interface is to facilitate PINK1 stabilization at the TOM complex following mitochondrial depolarization.

### 3.5. Decreased stabilization and activation of endogenous PINK1 in homozygous *PINK1^Q126P^* patient-derived fibroblasts

**Figure 5.**
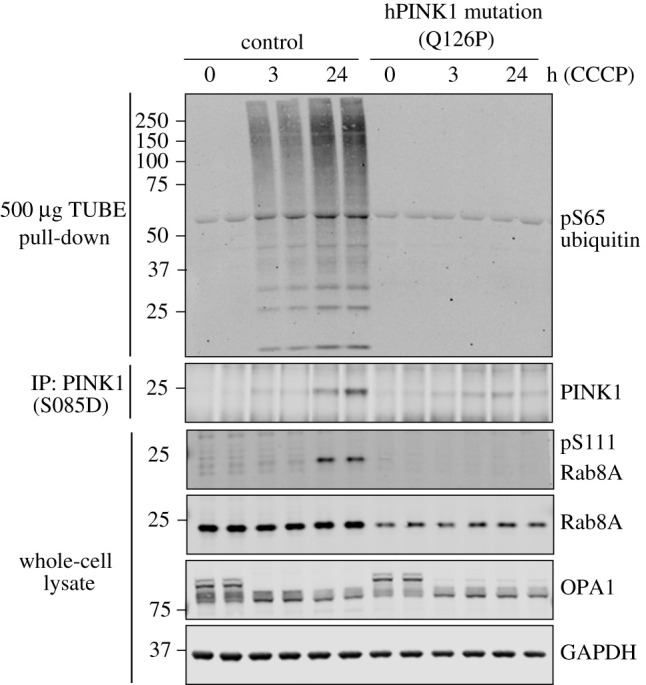
Endogenous PINK1 stabilization and activation is reduced in Q126P PINK1 patient-derived fibroblasts. Primary fibroblast cultures established from skin biopsies from a healthy subject (control) and a PD patient harbouring a PINK1 Q126P homozygous mutation were treated with 10 μM CCCP or DMSO for 3 h or 24 h. Lysates were subjected to immunoblot analysis using the indicated antibodies. Ubiquitin capture was used prior to immunoblotting with anti-phospho-Ser65 ubiquitin antibody. Endogenous PINK1 was detected after immunoprecipitation from whole-cell lysate.

We previously identified two sisters carrying homozygous mutations in PINK1, p. Q126P [15]. Both sisters manifested with early-onset PD at the age of 37 and 41 years and were longitudinally assessed in the dopaminergic ON state at the outpatient clinic for PD, University Hospital Tuebingen. At the most recent follow-up, the disease duration was 39 and 30 years, respectively. Both sisters had only mild motor impairment (UPDRS-III: 15 and 20 points after 27 and 30 years disease duration) with good Levodopa response. Cognitive function was mildly impaired with 21 and 23 points in MoCA testing after 27 and 30 years disease duration. No manifest mood disturbances were seen (BDI 11 and 10 points after 27 and 30 years disease duration). In line with the benign disease course, CSF levels of Amyloid-beta_1_42_ (729 and 678 pg ml^−1^; cut-off ≥600 pg ml^−1^), total-Tau (159 and 196 pg ml^−1^; cut-off ≤450 pg ml^−1^), phosphorylated-Tau (40 and 37 pg ml^−1^; cut-off ≤60 pg ml^−1^) and NFL (640 and 598 pg ml^−1^; cut-off ≤966 pg ml^−1^) were normal after 26 and 29 years disease duration. Both sister showed no α-synuclein seeding activity using RT-QuIC assay. Primary skin fibroblast cultures of one sister with homozygous PINK1 Q126P mutation, and unaffected human control fibroblasts, were treated with 10 µM CCCP or DMSO for 3 h or 24 h (figure 5). Immunoprecipitation-immunoblotting with anti-PINK1 antibody demonstrated time-dependent stabilization of PINK1 in control fibroblasts but this was significantly reduced in fibroblasts expressing Q126P PINK1 (figure 5). This was associated with complete loss of phospho-ubiquitin and pSer111-Rab8A in Q126P fibroblasts compared to control cells, indicating reduced PINK1 activation (figure 5).

## 4. Discussion

Combining mutational analysis with bioinformatic structural predictions of human PINK1, we have been able to map and elaborate a functional role for an N-terminal α-helical extension (NTE domain) of human PINK1 (figures 2 and 3; electronic supplementary material, figure S2). We demonstrate a critical role of the interaction of the NTE domain with the CTE domain, towards PINK1 stabilization, Ser228 autophosphorylation and activation, and that human disease-causing mutations of PINK1 that lie within the NTE–CTE interface disrupt PINK1 activation (figure 4; electronic supplementary material, figure S10B). Finally, we report the impact of homozygous NTE mutation, Q126P, on preventing stabilization and activation of endogenous PINK1 in patient-derived fibroblasts (figure 5).

PINK1 stabilization is regulated by multiple factors, notably the abrogation of proteolysis by PARL and other proteases upon mitochondrial depolarization [34]. Active PINK1 is stabilized in the TOM complex [11,12]; however, to date the molecular mechanism of how this contributes to PINK1 activation remain to be fully elucidated. Interestingly, we observed that the HSP60-MTS (residues 1–27) and PINK1 MTS (residues 1–34) both permit PINK1 import; however, the PINK1 MTS did not lead to PINK1 activation. In future work, it will be interesting to understand whether the mitochondrial import mechanism of PINK1 MTS and HSP60-MTS-PINK1 is different, or whether they are both imported via the TOM complex but their association with it upon mitochondrial depolarization is distinct.

Previous analysis of PINK1 mutants have found that disease mutants that affect intrinsic catalytic activity disrupt PINK1 stabilization at the TOM complex, and this is consistent with our observations with kinase-inactive and ATP-binding-defective mutants of PINK1 (figure 4) [11,12]. The finding that NTE or CTE domain mutants have similar impacts on PINK1 stabilization at the TOM complex independent of intrinsic catalytic activity provide new insights into PINK1 stabilization (figures 4 and 5; electronic supplementary material, figure S10B). Studies in TOM7 knockout cells have demonstrated that TOM7 is essential for PINK1 kinase activation [26]. A recently uploaded preprint has described the structure of *Tribolium castaneum* PINK1 (TcPINK1) that provides direct experimental evidence for the NTE : CTE molecular interaction [35]. Future structural studies will be required to determine whether the NTE : CTE interface facilitates PINK1 binding directly to TOM7.

Interestingly, we observed that substrate binding-defective mutants, G309D and G409 V, that exhibit robust autophosphorylation at Ser228, stabilize as well as WT PINK1 (figure 4). This suggests that autophosphorylation of PINK1 rather than substrate phosphorylation of PINK1 contributes to PINK1 stabilization. We found that the S228A mutant exhibited impaired stabilization at the TOM complex similar to kinase-inactive PINK1 (electronic supplementary material, figure S10B), in contrast with a previous study [12], suggesting that PINK1 autophosphorylation at Ser228 is the major site mediating PINK1 stabilization at the TOM complex.

Our cell-based analysis of disease-associated mutants of PINK1 using protein stabilization, substrate-based readouts and autophosphorylation reveals quantitative differences between ATP-binding mutants (A168P, E240 K); magnesium-binding mutants (D384A); substrate-binding mutants (G309D, G409 V) and NTE–CTE interface mutants (figure 4*a*). This could serve as robust framework to evaluate the functional impact of novel PINK1 mutations in the future and determine whether they can be classified within these four broad functional groups.

In that regard, we did not observe any biochemical defect of the L539F mutation on PINK1 activation (figure 4). This mutation has been reported in only one patient/family with PD compared to the other mutations we assessed which have been found in multiple independent families, and overall our data indicates that this mutation is not pathogenic and unlikely to be the cause of Parkinson's in the reported case [28]. By contrast, the clinical relevance and importance of the function of the NTE : CTE interface is supported by the demonstration of defective endogenous PINK1 stabilization and activation in patient-derived fibroblasts of a homozygous Q126P mutation. The age of onset of the patient was less than 45 years and the slow progression is clinically consistent with PINK1 pathogenic mutations in humans. Identifying further patients harbouring pathogenic mutations at the NTE : CTE interface will be important to aid in translational studies exploiting this regulatory mechanism of PINK1.

**Figure 6.**
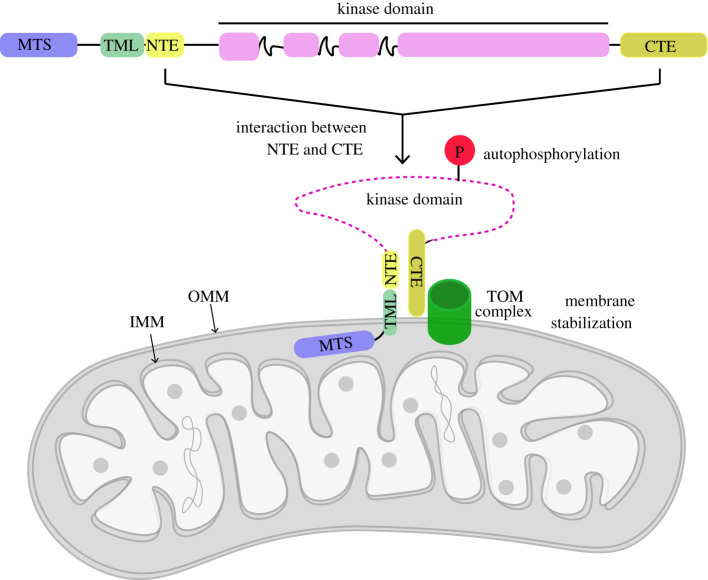
Schematic of role of NTE : CTE interaction towards PINK1 stabilization at mitochondria. NTE domain interaction with CTE facilitates stabilization and activation of PINK1 at TOM complex. Abbreviations: mitochondrial-targeting sequence (MTS); transmembrane-like domain (TML); N-terminal extension domain (NTE); C-terminal extension (CTE); translocase of outer membrane (TOM); inner mitochondrial membrane (IMM); outer mitochondrial membrane (OMM).

In conclusion, our studies have revealed an important functional role for the NTE α-helical domain of PINK1 and its interaction with the CTE as a mechanism required for PINK1 stabilization at the TOM complex (figure 6). Our findings suggest that small molecules that target the NTE : CTE interface to enhance association with the TOM complex could have therapeutic benefit in select PINK1 patients, and the feasibility of this approach will be aided by further structural analysis.

## Data accessibility

All primary data associated with each figure are available from Zenodo: https://doi.org/10.5281/zenodo.5767328. The HDX mass spectrometry proteomics data have been deposited to the ProteomeXchange Consortium via the PRIDE partner repository with the dataset identifier PXD030382 (username: reviewer_pxd030382@ebi.ac.uk; password: AH2OmZop) [36].
